# Special Section Guest Editorial: Biophotonics in Global Health

**DOI:** 10.1117/1.BIOS.2.4.042301

**Published:** 2026-01-01

**Authors:** Vanderlei Bagnato, Rebecca Richards-Kortum, Jenna Mueller

**Affiliations:** a Texas A&M University, College Station, Texas, United States; b University of São Paulo, São Paulo, Brazil; c Rice University, Houston, Texas, United States; d University of Maryland, College Park, Maryland, United States

## Abstract

Guest Editors Rebecca Richards-Kortum, Vanderlei Bagnatpo, and Jenna Mueller introduce the Special Section on Biophotonics in Global Health.

Optics in general has long played a key role in driving solutions to a wide range of health-related challenges. Although human health has been a major area of focus, the impact of optical technologies extends far beyond that. Places like environmental monitoring, animal health, and other fields have also greatly benefited from modern optical techniques used in diagnosis and even treatment through the application of light-based tools.

The rapid advancement of biomedical engineering, together with physics and chemistry, has enabled remarkable progress in disease diagnosis and treatment. We have never understood so well how to detect and manage conditions such as cancer and chronic diseases like Alzheimer’s, Parkinson’s, and osteoarthritis. These accomplishments have been driven by increasingly precise and sophisticated technologies.

Paradoxically, however, these very technologies have never been so inaccessible to the general population. Their high cost places them beyond the economic reach of much of society. While new technologies are undeniably a powerful force transforming human health, they often lack the level of accessibility that is urgently needed.

Global health surveys clearly reveal a substantial gap between high-income and low-income countries regarding the adoption of modern technologies. Achieving meaningful progress in global health requires that we consider cost as a fundamental factor and direct our efforts toward developing technologies that can genuinely address health challenges while remaining financially viable for most countries. In some nations, annual per-capita healthcare spending surpasses US$5,000, while in others it does not reach US$10. Addressing this disparity is a responsibility shared by all, and biophotonics offers possibilities that few other fields can.

This *BIOS* Special Section on Biophotonics in Global Health aims to inspire students, professionals, and scientists to apply their expertise toward this pressing need. In this issue, we present devices that use wide-field or fluorescence imaging to enable diagnosis and even treatment assessment across various medical applications. From smartphone-based systems to intra-oral examination devices and tools for sample preparation, this collection of articles opens an important discussion. We hope that this journal will continue to receive many contributions on this vital topic in the years to come.

In this collection case, the articles clearly reflect their intended spirit. “Optimization of a mobile imaging system to evaluate patients with oral lesions in a dental care setting” (doi 10.1117/1.BIOS.2.4.042307), by Ruchika Mitbander and coauthors, presents a system designed to improve the clarity, accuracy, and consistency of images captured during dental examinations. Adjustments to lighting, color balance, and resolution make the process more efficient and accessible for clinics with limited infrastructure. Because the system is mobile, it broadens diagnostic reach, supporting early detection of oral diseases in diverse environments and contributing to more reliable clinical decisions and improved patient outcomes. “*In vivo* fundus imaging and computational refocusing with a diffuser-based fundus camera” (doi 10.1117/1.BIOS.2.4.042306), by Corey Simmerer and coauthors), explores an optical approach that uses a thin diffuser to capture multiplexed retinal information without relying on complex ophthalmoscopic systems. The coded pattern generated by the diffuser enables computational refocusing after acquisition, offering a low-cost and potentially portable solution for enhanced retinal diagnostics in point-of-care settings. “Enabling point-of-care optical diagnostics and treatment of oral lesions in resource-limited settings” (doi 10.1117/1.BIOS.2.4.042305), by Shakir Khan and collaborators, describes the development of an affordable, portable system that detects and treats oral lesions on a single platform. The device integrates real-time optical imaging with optimized light delivery for photodynamic therapy, enabling precise and minimally invasive intervention. This therapeutic approach aims to expand access to early diagnosis and treatment of oral diseases. “Low-cost optical system for laser phacoemulsification of cataracts” (doi 10.1117/1.BIOS.2.2.022304), by Mitchell Harrah et al., introduces a simplified and affordable laser-based platform designed to fragment and remove cataracts with greater precision and less mechanical trauma than traditional ultrasonic phacoemulsification. Using cost-effective optical components and compact laser sources, the system emphasizes portability, reliability, and ease of operation, expanding access to cataract surgery in underserved regions. “Radiomic identification of anemia features in monochromatic conjunctiva photographs in school-age children” (doi 10.1117/1.BIOS.2.2.022303), by Shaun Hong et al., applies radiomic analysis to detect subtle markers of anemia from standardized conjunctival images. By extracting texture, intensity, and spatial features, the method enhances sensitivity to visual cues that are not easily perceived by clinicians, offering potential for extensive-scale screening in resource-limited environments. Finally, “Improved performance and design of a low-cost laparoscope to enable laparoscopic surgery in low-income countries” (doi 10.1117/1.BIOS.2.2.022302), by Anne Christine Barnes and coauthors, demonstrates advances in the development of an affordable laparoscope capable of delivering imaging quality and usability comparable to standard surgical tools. Improvements in optical design, illumination efficiency, and sensor selection aim to provide clear intraoperative images using low-cost components, expanding access to laparoscopic surgery and reducing postoperative complications in resource-constrained hospitals.

